# Dosimetry in cranial photobiomodulation therapy: effect of cranial thickness and bone density

**DOI:** 10.1007/s10103-024-04024-z

**Published:** 2024-02-22

**Authors:** Sergio Castaño-Castaño, Candela Zorzo, Juan Á. Martínez-Esteban, Jorge L. Arias

**Affiliations:** 1 Department of Psychology, University of Oviedo, 33003 Oviedo, Spain; 2 Neuroscience Institute of the Principality of Asturias (INEUROPA), Oviedo, Spain; 3 Health Research Institute of the Principality of Asturias (ISPA), Oviedo, Spain; 4 Department of Electrical Engineering, Computer Electronics, and Systems, Polytechnic School of Engineering, University of Oviedo, 33203 Gijón, Spain

**Keywords:** Photeraphy, Skull, Linear model, Logarithmic model, Laser

## Abstract

This research aims to examine the influence of human skull bone thickness and density on light penetration in PBM therapy across different wavelengths, focusing on how these bone characteristics affect the absorption of therapeutic light. Analyses explored the effect of skull bone density and thickness on light penetration in PBM, specifically using Low-Level Laser Therapy (LLLT) for efficacy prediction. Measurements of bone thickness and density were taken using precise tools. This approach emphasizes LLLT's significance in enhancing PBM outcomes by assessing how bone characteristics influence light penetration. The study revealed no significant correlation between skull bone density and thickness and light penetration capability in photobiomodulation (PBM) therapy, challenging initial expectations. Wavelengths of 405 nm and 665 nm showed stronger correlations with bone density, suggesting a significant yet weak impact. Conversely, wavelengths of 532 nm, 785 nm, 810 nm, 830 nm, 980 nm, and 1064 nm showed low correlations, indicating minimal impact from bone density variations. However, data variability (R^2^ < 0.4) suggests that neither density nor thickness robustly predicts light power traversing the bone, indicating penetration capability might be more influenced by bone thickness at certain wavelengths. The study finds that the effectiveness of photobiomodulation (PBM) therapy with bone isn't just based on bone density and thickness but involves a complex interplay of factors. These include the bone's chemical and mineral composition, light's wavelength and energy dose, treatment duration and frequency, and the precise location where light is applied on the skull.

## Introduction

Currently, non-invasive brain stimulation techniques are proving their therapeutic efficacy, and their applications are expanding to treat various neurological, psychiatric, or psychological disorders. Among them is photobiomodulation (PBM) therapy. This technique has been used for a wide range of disorders affecting the nervous system, such as for the treatment of traumatic events like stroke or traumatic brain injury, to intervene in degenerative diseases and psychiatric or psychological disorders, and even to prevent cognitive decline in healthy aging [1–5].

PBM employs the direct application of low-intensity red or near-infrared light as well as high-power lasers (Class IV lasers), and can also be implemented using light-emitting diodes (LED), representing another effective modality within the spectrum of photobiomodulation.Its action mechanism is based on its effect on the molecular chromophores present in our cells. PBM emits photons absorbed by these chromophores, which can activate specific signaling pathways and transcription factors that can induce changes in the expression of specific proteins, optimizing the functioning of nerve cells [6]. However, it is essential to consider that the interaction between light and biological tissues can vary widely because it depends on different wavelengths, frequency, power, and the characteristics of the biological tissue to which light is applied [7].

Since its first use in 1968, photobiomodulation has been shown to promote hair growth and wound healing in rats [8]. Today, it is understood that PBM's effects act on the mitochondrial respiratory chain enzyme, a photoreceptor that absorbs specific wavelengths, leading to molecular and cellular changes, and activating protective, antioxidant, and anti-apoptotic signaling pathways [6]. Mitochondria's photon absorption capacity ranges from 600 to 1,100 nm, with absorption peaks in red (620–689 nm) and infrared (760–825 nm) spectra, and longer wavelengths like 1064–1072 nm enhance cerebral oxygenation [9, 10]. Most clinical and experimental studies employ wavelengths of 800, 810, or 1064 nm, though shorter wavelengths like 660 nm, or longer ones like 1072 nm, have also been used [2].

To achieve a neuroprotective effect, PBM must overcome several barriers, including bone tissue, a complex structure with variable optical properties [11]. Previous studies have shown that near-infrared light can efficiently penetrate bone, important for imaging techniques and light-based therapies like PBM [12, 13]. Light penetration, influenced by wavelength, intensity, irradiance, and bone density and composition, as well as tissue optical properties, is crucial [14]. A significant portion of light can penetrate outer layers and reach nervous tissue, with up to 10, 20, and 30 mm penetration in the brain, and in some cases, up to 50 mm and 2 cm, extendable to 3 cm with combined emission parameters [3, 15, 16]. Jagdeo et al.'s study [14] examined the transmission of red and near-infrared light through soft tissues, blood, brain parenchyma, and the skull, considering thickness and density. They found that while blood attenuates light transmission, extensive infrared light transmission occurs through soft tissues, and near-infrared penetrates these barriers, including the skull and brain parenchyma, more effectively than red light [13]. Additionally, the penetrance of photobiomodular therapy has been explored about skull and scalp thickness and in different skull areas. It was discovered that light penetrance in the brain decreases with age due to increased brain size and extracerebral layer thickness and that the 810 nm wavelength offers the highest energy deposition, with 850 and 1064 nm wavelengths offering more than 670 and 980 nm [11].

Despite these promising advances, a detailed understanding of how light penetration capabilities vary depending on wavelength and other parameters remains limited. Some studies have found that longer wavelengths can penetrate deeper into bone tissue, but these results have been inconsistent and are based on bone tissue models that do not fully capture its structural complexity and composition [17, 18]. This variability and lack of a comprehensive understanding of light penetration in bone tissue present a challenge in the application of PBM. As can be seen from the above, a detailed understanding of how the optical properties of bone tissue affect light penetration is essential to improve the effectiveness of PBM and optimize its use as a potential treatment.

Therefore, the aim of this work, conducted as an experimental study, is to analyze the relationship between the thickness and density of the human skull bone and how this influences light penetration, considering various wavelengths from near-ultraviolet to near-infrared. In this experimental approach, we actively manipulate and assess the impact of different light wavelengths on bone penetration, providing a controlled evaluation of these variables' interplay.

## Method

### Sample

A total of 25 adult human cranial vaults from the Faculty of Medicine at the University of Oviedo (Spain) were used. From each skull, two sample pieces measuring 1.5 × 1.5 cm were obtained from the right (Fp2) and left (Fp1) supraorbital region, yielding a total of 50 samples (*n* = 50).

The entire procedure followed the strictest quality control measures and respected the ethical guidelines for research with human samples (Law 14/2007, of July 3, on Biomedical Research, Royal Decree 1716/2011, Directive 2004/23/EC).

### Devices

In this study, the researchers utilized a collection of photobiomodulation devices composed of advanced laser diodes (Osram opto, Thorlabs), each tailored to emit a specific wavelength. These devices are adept at producing light across a spectrum ranging from near-ultraviolet (405 nm) to near-infrared (1064 nm), with specific wavelengths including 405 nm (GaN), 532 nm & 1064 nm (Nd:YAG), 655 nm, 780 nm, 810 nm, & 830 nm (GaAs), and 980 nm (InGaAs). The emitted powers for each wavelength are the maximum emitted by each diode: 27,100 μW (0.0271 W), 81,000 μW (0.081 W), 66,000 μW (0.066 W), 57,700 μW (0.0577 W), 113,800 μW (0.1138 W), 60,700 μW (0.0607 W), and 176,200 μW (0.1762 W), showcasing the devices' capability to cover a broad range of therapeutic needs by adjusting for bone density and thickness variations, respectively. The diodes in the study continuously emitted light, key for understanding light absorption and penetration in bone tissue. Covering wavelengths from near-ultraviolet (405 nm) to near-infrared (1064 nm) and emitted powers from 27,100 μW to 176,200 μW, they allow comprehensive investigation into how various wavelengths penetrate bone. Each laser diode is specialized for its wavelength, ensuring consistent, pure light emission, vital for accurately assessing each wavelength's impact on bone penetration in the experiments.

To evaluate the power of the light that passed through the skull bone samples, a ThorLabs PM160 optical power meter was used. This device features a photodiode sensor, designed to measure optical power in a wavelength range of 400 to 1100 nm, suitable for the variety of laser diodes used in this study. The meter has an optical power measurement range of 10 nW to 2 mW, extendable up to 200 mW with a sliding neutral density (ND) filter. The measurement resolution for optical power is 100 pW, which can increase up to 10 nW with the ND filter. The sensor's design includes an ultra-thin sensor end of 3.5 mm thickness (6 mm with the ND filter), allowing for precise placement and minimization of potential measurement interferences. The sensor aperture is Ø9.5 mm, and the device comes with an SM05 optical fiber adapter.

To obtain and analyze optical power measurements, the PM160 GUI version 1.1.0 software was used. This software, developed by ThorLabs, is designed to work seamlessly with the PM160 optical power meter.

To optimize the measurement process and ensure consistency and accuracy of the results, a custom mounting device was designed and fabricated using 3D printing technology. This device, consisting of a base on which the ThorLabs PM160 optical power meter (non-invasive and validated instrument) was placed and an upper part designed to house the different laser diodes, allowed for standardized and controlled measurements. The device was specifically designed to hold the bone samples on its base while the various laser diodes used in the measurements were placed on the top. The dimensions of the device, such as height, width, and depth, were carefully determined to ensure a precise fit of the bone samples and the correct placement of the lasers.

The use of this custom mounting device ensured that the light beam from each laser diode was applied perpendicularly and centered on the surface of the power meter, minimizing potential variations in measurements due to the orientation or positioning of the laser.

### Measurement procedure

The measurement procedure to determine the power of light passing through the cranial bone samples was conducted on 1.5 cm × 1.5 cm samples from each skull at the FP1 and FP2 regions, following the international 10–20 system of EEG.

To measure bone thickness, we employed a digital caliper with a resolution of 0.01 mm, ensuring precise measurements of bone thickness. The technique involved applying the caliper at predetermined, consistent points on each bone sample, recording multiple measurements for reliability. As for bone density, we implemented a water displacement method. Each bone sample was submerged in a graduated cylinder filled with water, noting the change in liquid volume. Density was subsequently calculated using the density formula (mass/volume), where mass was determined using a precision scale. These methods provide a detailed and technical analysis of the bone's physical properties.

Bone samples were placed on the base of the 3D-printed mounting device, directly above the ThorLabs PM160 optical power meter. The different laser diodes were positioned on the top of the device, from where the light beam was emitted onto the marked point on the bone sample. Each bone sample was given a specific orientation, and a specific point was marked on which the light from the different lasers used in the test would be incident. This standardization ensured that all measurements were carried out under the same conditions, minimizing any variability that might influence the results. Upon emitting the light, the meter located below the bone sample recorded the power of the light that managed to pass through it. The wavelengths used were 405 nm, 532 nm, 655 nm, 780 nm, 810 nm, 830 nm, 980 nm, and 1064 nm. These measurements were carried out in a controlled environment in the laboratory, ensuring reproducible conditions for each test. The evaluator responsible for measuring optical power was indeed blinded during the study. Each sample was numbered, but the evaluator did not have precise information regarding the thickness and density of the samples, ensuring an unbiased assessment of light power passing through the bone.

### Statistics

In their study, the researchers evaluated the relationship between bone density and thickness using Spearman's correlation, chosen due to its suitability for non-normally distributed data, as confirmed by the Shapiro–Wilk test. Spearman's correlation was used to measure both linear and non-linear monotonic relationships, fitting the potential non-linear nature of their biological data.

The interpretation of the R values was conducted according to the following scheme [19]:

- R = 1: Perfect correlation. Indicates a direct and proportional relationship between the variables.
- R = 0.9 < 1: Excellent. Shows a very strong association between the variables.
- R = 0.8 < 0.9: Good. Reflects a strong and significant relationship.
- R = 0.5 < 0.8: Fair. Represents a moderate correlation, which may be relevant but not as strong.
- R < 0.5: Poor. Indicates a weak correlation, suggesting a less significant or nonexistent relationship between the variables.

This classification allows us to better interpret the relevance and strength of the associations found in our study, providing a useful guide for understanding the interaction between the analyzed variables.

In our linear regression studies, we employed Spearman's correlation coefficient (Rho) to assess the strength of the linear relationship between variables. The interpretation of Rho values was based on the following criteria [19]:

- Rho = 1 to 0.7: Strong linear relationship. Indicates a high degree of association between the variables.
- Rho = 0.3 to 0.7: Moderate. Reflects a moderate degree of association.
- Rho = 0.1 to 0.3: Weak. Suggests a low, but potentially significant, level of association.
- Rho = 0 to 0.1: None. Implies no significant linear relationship between the variables.

Additionally, linear regression analyses were performed for each laser wavelength to explore the relationship between laser power and bone density and thickness. To further address non-linear relationships indicated by Spearman's correlation, logarithmic regression analyses were conducted.

In our logarithmic regression models, we used the coefficient of determination (R^2^) to assess how well the model explains the variability of the data. The interpretation of R^2^ values was based on the following criteria [19]:

- R^2^ = 1 to 0.9: Explains the variability of the data. Indicates that the model accounts for almost all of the variability in the response data around its mean.
- R^2^ = 0.7 to 0.9: Explains a significant amount of the variability. Reflects that the model accounts for a substantial part of the variability in the response data.
- R^2^ = 0.4 to 0.6: Moderate fit. Indicates that the model does not explain a significant portion of the variability in the data.
- R^2^ < 0.4: Does not explain the variability of the data. Suggests that the model fails to account for a considerable part of the variability in the response data.

This classification helps in evaluating the effectiveness of our logarithmic regression models in explaining the observed data, providing a clear framework for interpreting the results.

This combination of statistical methods provided a comprehensive examination of the data. All analyses were done using IBM SPSS Statistics 27.0, considering *p* ≤ 0.05 as the threshold for statistical significance, in line with standard scientific practices.

## Results

In the analysis of our data, the Shapiro–Wilk test was conducted to assess the normality of our two main variables: skull bone thickness and density. In the case of bone thickness, a Shapiro–Wilk statistic of 0.965 (df = 50, *p* = 0.137) was obtained. This result was not significant (*p* > 0.05), indicating that we can assume a normal distribution for the bone thickness data. In contrast, for bone density, a Shapiro–Wilk statistic of 0.946 (df = 50, *p* = 0.024) was obtained. This result was significant (*p* < 0.05), suggesting that the bone density data do not follow a normal distribution. It's important to note that when we want to correlate two samples, and one follows a normal distribution while the other does not, we should use a non-parametric correlation test.

Upon analyzing the obtained samples, no significant correlation was found between the thickness and density of the cranial bone (*Rxy* = 0.07, *p* = 0.629). When considering non-parametric correlations in our study, we used Spearman's correlation coefficient (R) to assess the strength and direction of the association between variables.

**Fig. 1:**
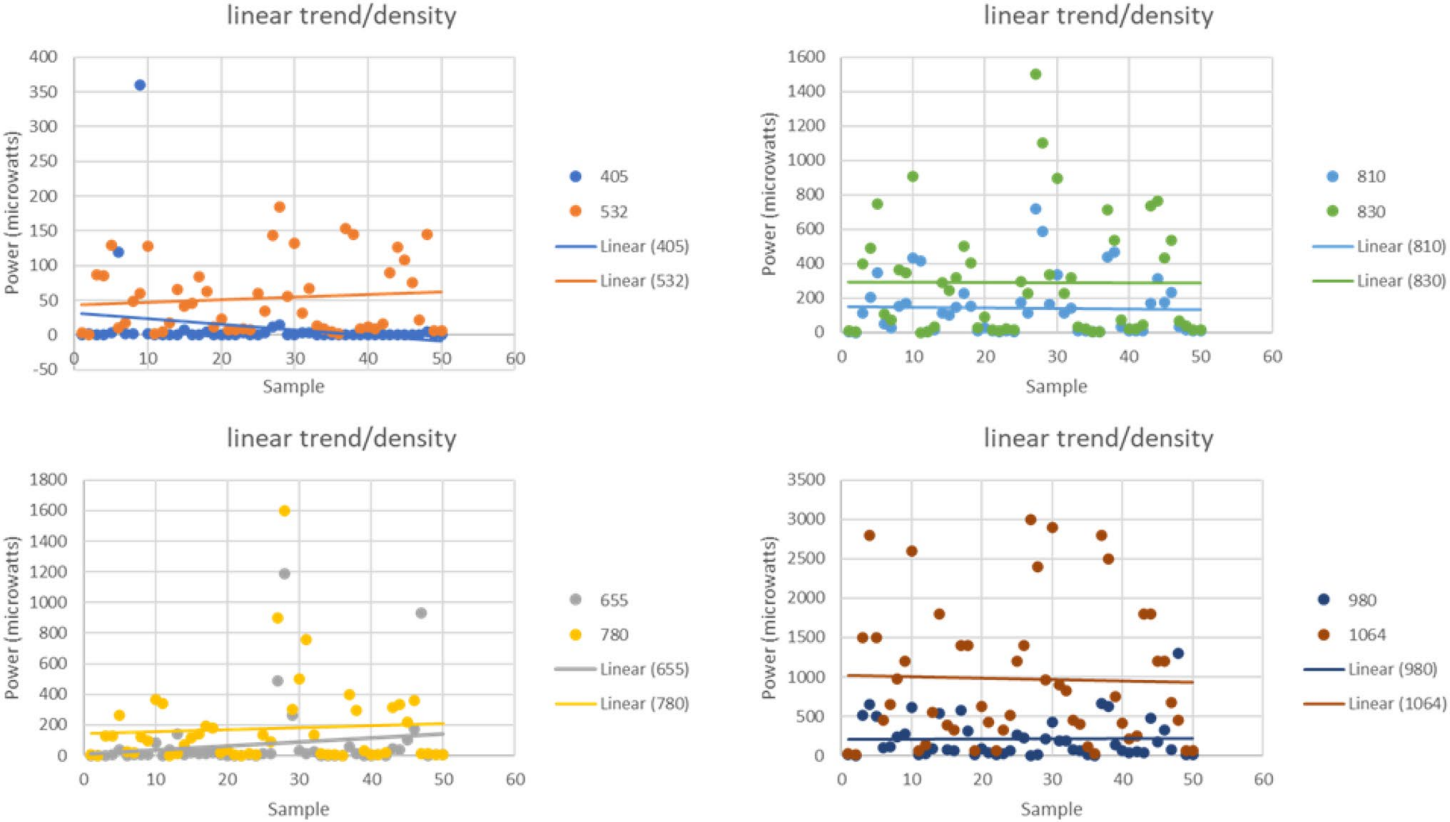
Wavelengths of 405 nm (R = 0.343, *p* = 0.015) and 665 nm (R = 0.293, *p* = 0.039) demonstrate a weak albeit significant linear relationship with bone density. In contrast, wavelengths ranging from 532 to 1064 nm, such as 1064 nm (R = 0.77, *p* = 0.596) and 785 nm (R = 0.033, *p* = 0.819), show no relevant correlation, suggesting a minimal impact on bone density

Linear regression studies, taking bone density into account, showed the strongest correlations with lasers of 405 nm (*R* = 0.343, *p* = 0.015) and 665 nm (*R* = 0.293, *p* = 0.039). This suggests that these wavelengths have a significant effect on bone density, establishing a weak linear relationship. In contrast, wavelengths of 1064 nm (*R* = 0.77, *p* = 0.596), 980 nm (*R* = 0.153, *p* = 0.291), 830 nm (*R* = 0.089, *p* = 0.540), 810 nm (*R* = 0.094, *p* = 0.514), 785 nm (*R* = 0.033, *p* = 0.819), and 532 nm (*R* = 0.15, *p* = 0.917) displayed the lowest correlations. This indicates that bone density has a minimal impact on the wavelengths passing through it, with a virtually non-existent linear relationship (Fig. 1).

Regarding the relationship between the power of light that penetrates the bone and the thickness of the bone, the wavelengths of 532 nm (R = 0.590, *p* = 0.000), 830 nm (R = 0.499, *p* = 0.000), 980 nm (R = 0.481, *p* = 0.000), and 1064 nm (R = 0.603, *p* = 0.000) had the highest correlations with bone thickness. This suggests that bone thickness might have a more significant impact on these wavelengths, given the high significance, even though the predictive model shows moderate values.

**Fig. 2:**
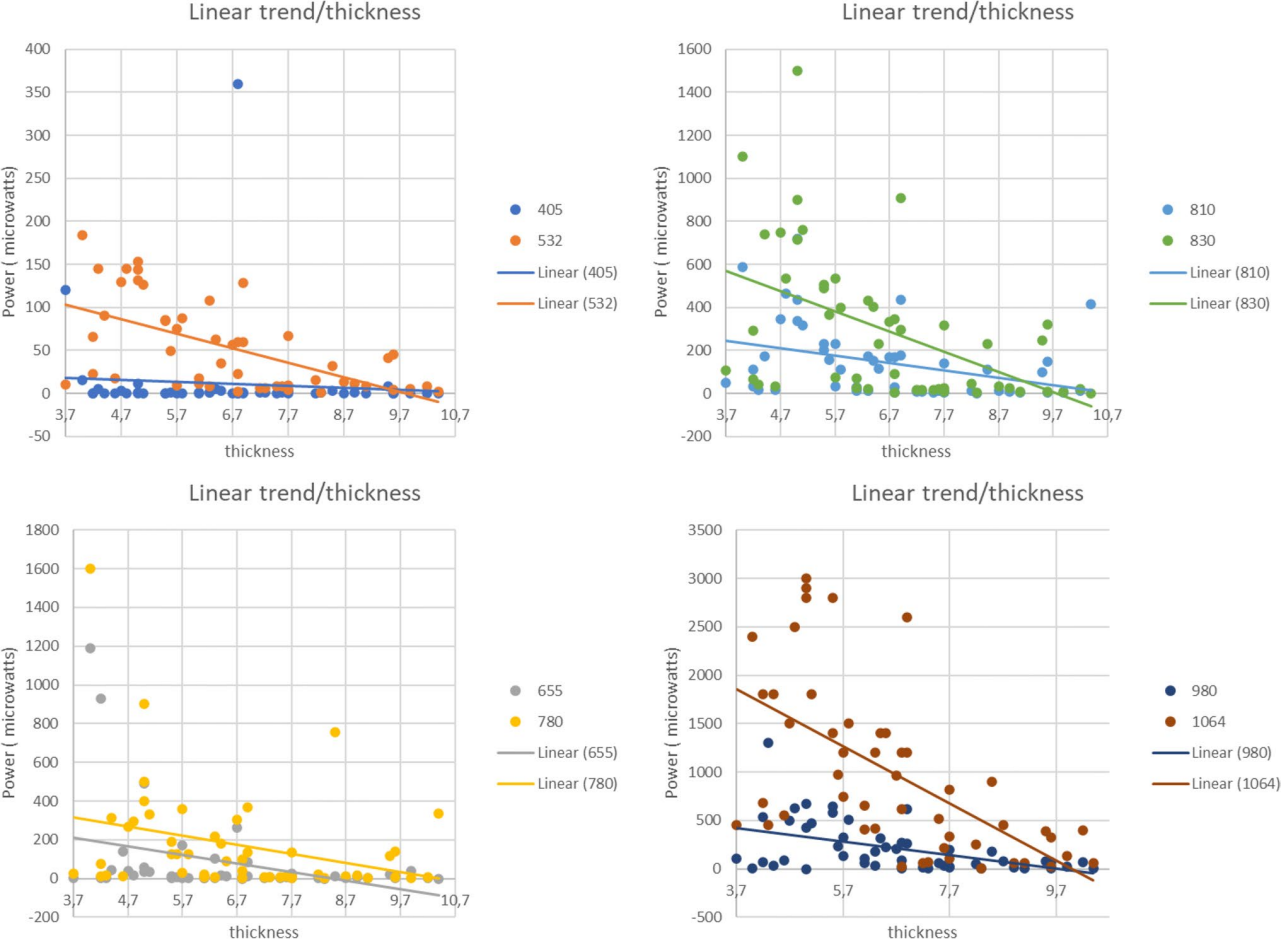
The wavelengths of 532 nm, 830 nm, 980 nm, and 1064 nm show high and significant correlations with bone thickness, indicating its notable impact on those wavelengths. The wavelengths of 665 nm, 780 nm, and 810 nm have lower albeit significant correlations with bone thickness. In contrast, the wavelength of 405 nm shows a weak and non-significant correlation, suggesting a minimal impact of bone thickness on this wavelength

On the other hand, lasers with wavelengths of 665 nm (R = 0.359, *p* = 0.010), 780 nm (R = 0.300, *p* = 0.035), and 810 nm (R = 0.364, *p* = 0.009) displayed the weakest correlation. Still, they indicated a significant effect when considering bone thickness. For the wavelength of 405 nm (R = 0.158, *p* = 0.237), the correlation value indicated a weak predictive model, suggesting that bone thickness has minimal impact on this wavelength (Fig. 2).

**Fig. 3:**
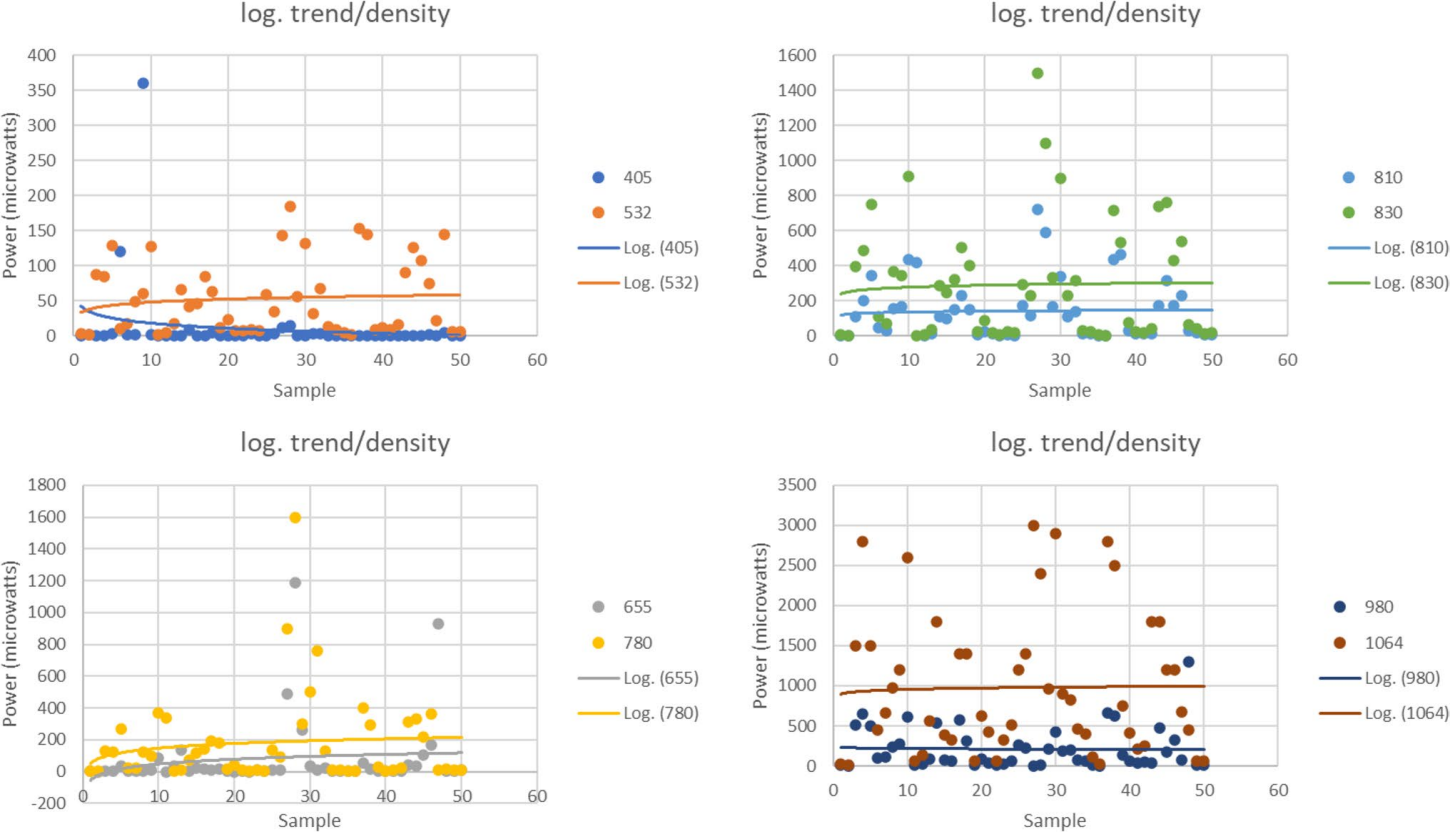
Logarithmic model where wavelengths of 450 nm and 665 nm show a significant impact of bone density on the power of light passing through it, with *p*-values < 0.05. In contrast, wavelengths ranging from 532 to 1064 nm do not exhibit a significant impact of bone density, as their *p*-values are > 0.05. The low variability (R2 < 0.4) in the data suggests that, within this model, bone density is not a robust predictor of the power of the light passing through it

The logarithmic regression studies with the light power passing through bone as an independent variable showed the following values for different wavelengths: 450 nm (R^2^ = 0.097, *p* = 0.028), 665 nm (R^2^ = 0.100, *p* = 0.025), suggesting that bone density has the most significant impact on these wavelengths in a logarithmic model (*p* < 0.05). On the other hand, the values obtained for wavelengths of 532 nm (R^2^ = 0.001, *p* = 0.870), 785 nm (R^2^ = 0.001, *p* = 0.848), 810 nm (R^2^ = 0.008, *p* = 0.522), 830 nm (R^2^ = 0.010, *p* = 0.492), 980 nm (R^2^ = 0.026, *p* = 0.262), and 1064 nm (R^2^ = 0.008, *p* = 0.536) suggest that bone density does not have a significant impact on these wavelengths in a logarithmic model (*p* > 0.05). The data variability (R2 < 0.4) for bone density suggests that, in a logarithmic model, it is not a predictor of the light power passing through it (Fig. 3).

**Fig. 4:**
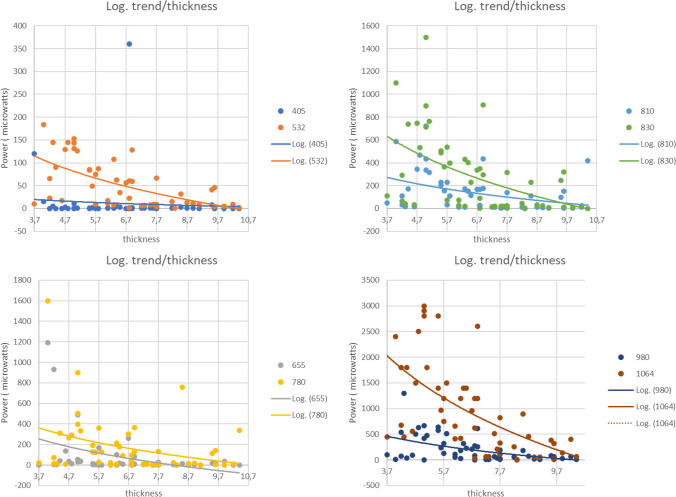
The wavelengths of 1064 nm, 830 nm, and 532 nm indicate a significantly high impact of bone thickness on the power of light passing through it in a logarithmic model (*p* < 0.001). The wavelengths of 665 nm, 785 nm, 810 nm, and 980 nm also show an impact in the logarithmic model, but with a lesser degree of significance (*p* < 0.05). However, the 450 nm wavelength does not appear to be significantly influenced by bone thickness in this model (*p* > 0.05). The overall variability (R2 < 0.4) suggests that bone thickness, in this model, is not a robust predictor of the power of the light passing through it

The results for the relationship between the power of the light passing through the bone and the thickness of the bone for the wavelengths 1064 nm (R2 = 0.349 *p* = 0.000), 830 nm (R2 = 0.247 *p* = 0.000), and 532 nm (R2 = 0.353 *p* = 0.000) suggest that these wavelengths might have a high impact considering the thickness of the bone in a logarithmic model (*p* < 0.001). Furthermore, values obtained in this regression for wavelengths 665 nm (R2 = 0.159 *p* = 0.004), 785 nm (R2 = 0.105 *p* = 0.022), 810 nm (R2 = 0.144 *p* = 0.007), and 980 nm (R2 = 0.224 *p* = 0.001) suggest that these wavelengths may have an impact considering the thickness of the bone in a logarithmic model (*p* < 0.05). The exception in this case is marked by the wavelength 450 nm (R2 = 0.029 *p* = 0.237), which seems to have no significant impact considering the thickness of the bone in a logarithmic model (*p* > 0.05). However, the variability of the data (R2 < 0.4) for bone thickness suggests that, in a logarithmic model, it is not a predictor of the power of the light passing through it (Fig. 4).

**Fig. 5:**
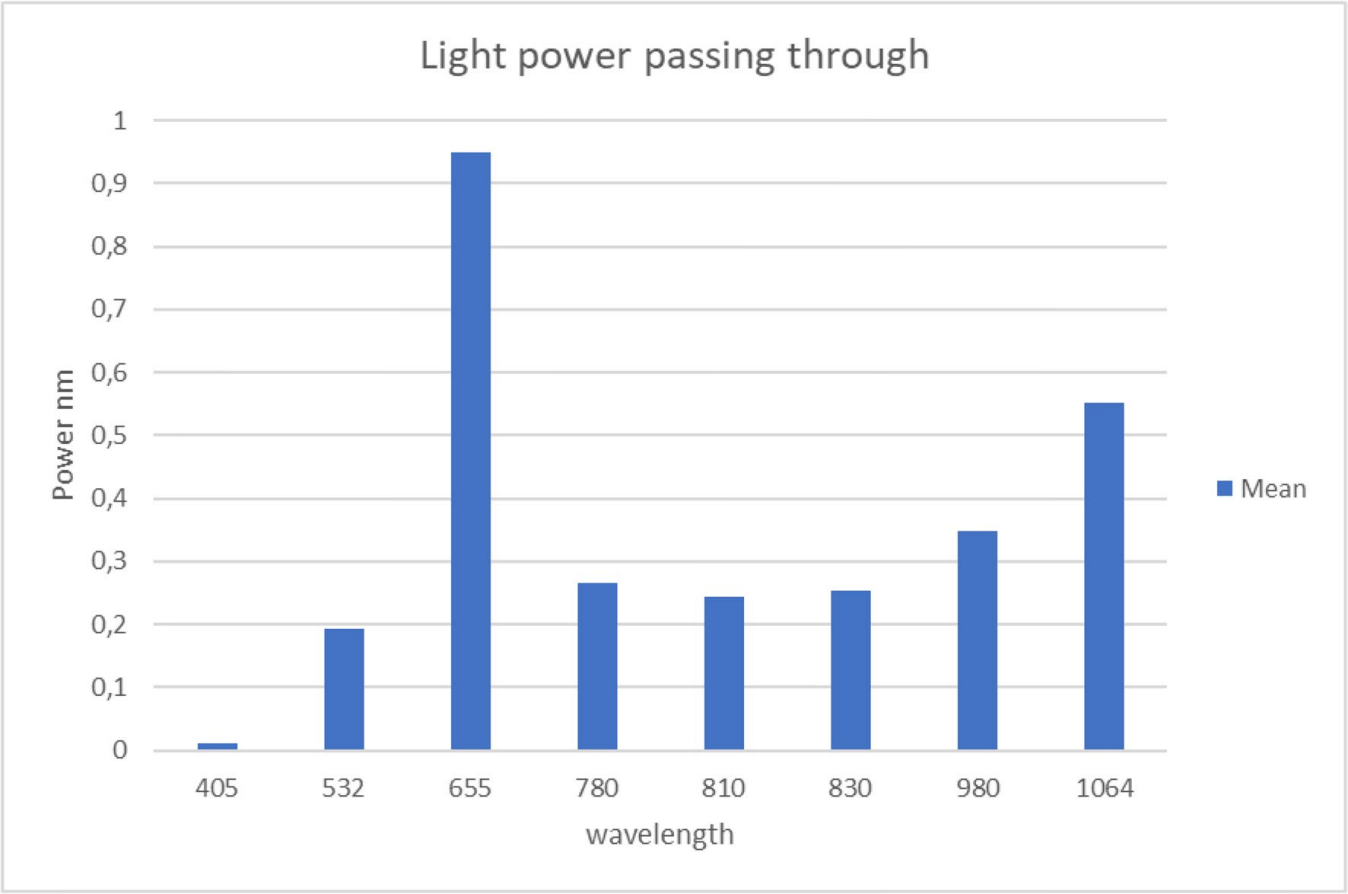
Graph of the power emitted in a vacuum by various laser diodes based on their wavelengths. The power passing through the bone is seen to vary depending on the wavelength. The 655 nm laser shows the highest average power passing through the bone at 0.95%, while the 405 nm laser shows the lowest at 0.01%

On the other hand, when considering the power emitted in a vacuum by the different laser diodes based on their wavelengths, the percentage of power that passes through the bone can be calculated for the various wavelengths. It was found that the highest average power for all samples was from the 655 nm laser at 0.95%, while the lowest power was from the 405 nm laser at 0.01% (see Fig. 5).

## Discussion

In the study, the relationship among three pivotal variables: the power of light penetrating cranial bones, bone density, and bone thickness was delved into using Photobiomodulation (PBM) devices that emit different wavelengths. Linear and logistic regression models were employed as the methodological approach, deemed effective for analyzing predictive relationships between dependent and independent variables [20].

The study focused on the right and left orbital frontal cortex, corresponding to Fp1 and Fp2, following the 10–20 electroencephalography system convention [21]. The selection of these areas for analysis was driven by the involvement of the frontal region of the brain, including the prefrontal cortex, in various cognitive and emotional functions [22–24].

The location of the orbital frontal cortex has emerged as a common target for non-invasive neuromodulation techniques, such as transcranial magnetic stimulation (TMS) and transcranial direct current stimulation (tDCS) [25, 26]. These techniques are particularly appealing due to their accessibility, safety, and empirical evidence of effectiveness in treating various neurological or psychological disorders [27–29]. Similarly, PBM is being used for treatment and rehabilitation following brain injury, in neurodegenerative diseases and psychiatric or psychological disorders [3–5]. It is becoming a promising intervention technique. PBM applications have achieved increased cerebrovascular oxygenation associated with neurocognitive improvement post-application [30], increased metabolic connectivity, and an effect on endothelial cells, causing changes in hemodynamic-metabolic coherence on each side of the prefrontal cortex [31]. Thus, most research suggests that PBM's beneficial effects stem from increased cerebral blood flow, greater availability and oxygen consumption, improved ATP production, and increased mitochondrial activity. Animal studies have shown that enduring changes might be due to the activation of particular signaling pathways and transcription factors, leading to shifts in the expression of specific proteins [3].

Photobiomodulation (PBM) differentiates itself from transcranial magnetic or electrical stimulation techniques in its mechanism of action, safety, and applicability. While PBM directly acts on cellular chromophores and enhances mitochondrial function, offering a more direct approach to treating neurological disorders, transcranial techniques modulate neuronal activity. PBM is characterized by high tolerability and minimal side effects, contrasting with potential complications of transcranial techniques. Moreover, its ease of use and portability surpass transcranial devices, which often require specific clinical settings. Although clinical evidence supports both modalities, PBM presents a promising field for future research due to its unique features [32].

In the context of photobiomodulation (PBM) for neurological disorders, choosing between LED and low-level laser therapy (LLLT) is crucial due to their inherent differences in light coherence, treatment precision, and penetration depth. Studies indicate that LLLT provides deeper penetration and precision, essential for targeting specific brain areas, while LED technology is advantageous for treating larger areas with a lower risk of thermal damage. Research suggests that wavelength and light coherence significantly influence treatment efficacy, emphasizing the importance of selecting the appropriate PBM type based on the specific condition being treated [15, 33].

In general, the results did not show a correlation between density and thickness, or a significant linear or logarithmic relationship between the laser's power penetrating the bone and the variables of density and thickness. This reveals the absence of a defined correlation between these variables in the studied sample. Therefore, neither cranial bone density nor thickness seem to play a crucial role in determining light penetration, suggesting the potential standardization of parameters. However, the study by Yuan et al. [11] observed that light penetrance in the brain decreases with the thickness of the extracerebral layers, among which the skull is included. Methodological differences with this study might explain our results, as that study encompasses both the skull and the scalp, while our study was restricted to the skull. Furthermore, Yuan et al. [11] included samples from birth to aging, which might translate into a more significant difference between the thicknesses of the skull and scalp, more homogeneous in an adult sample like in our study.

This leads us to postulate that other factors might play a more significant role in determining how much light can penetrate the cranial bone. The mineral and chemical composition of the bone is a factor that could have a substantial impact on light penetration. Bones are not homogenous; they are composed of an organic matrix and minerals such as calcium phosphate. This intricate framework can interact with light in various ways, altering its propagation [34]. Moreover, we must remember that bone is a dynamic tissue that is constantly remodeling throughout an individual's life. Likewise, factors like the orientation of trabeculae, the bone's water and fat content, and the presence of any bone damage or disease could influence light penetration. The absorption and scattering of light by the bone are complex processes that are influenced by multiple factors, and these might have a more significant impact than bone thickness and density [35].

The results of this study also show that a laser's wavelength is an important factor in determining its ability to penetrate bone. The 655 nm laser diode, with a wavelength in the near-infrared range, was the most effective, with 0.95% of its power penetrating the bone. In contrast, the 405 nm laser, with a wavelength in the ultraviolet range, had the lowest performance, with only 0.01% of its power managing to penetrate the bone.

These findings align with the results of previous studies showing that longer-wavelength lasers are more effective at penetrating bone tissue [14, 36, 37]. This is because longer-wavelength lasers possess lower photonic energy than shorter-wavelength lasers. This means they are less likely to interact with the bone tissue's atoms and molecules, allowing them to pass through more easily. As previously noted, the enhanced penetration of specific wavelengths over others might be due to the bone's intrinsic properties, such as its composition and structure, which could affect light absorption and scattering. Additionally, the variability in absorbing different wavelengths may result from differences in the amount of water, collagen, and other substances present in the bone.

Therefore, even though light penetration does not seem to directly depend on bone density or thickness, as per our regressions, there are differences between groups, suggesting other factors or combinations of factors might be at play.

Lastly, it must be considered that the effectiveness of PBM (Photobiomodulation) is determined not only by the chosen wavelength but also by the energy dose, duration, or frequency of treatment.. Given that there is no widely accepted set of optical properties for brain tissues, it is essential to continue research to determine the optimal light dosimetry for various treatments and conditions.

It is crucial to acknowledge the inherent limitations of our study. While we have investigated the relationship between cranial bone density and thickness and light penetration in PBM, our samples were exclusively derived from cadaveric skull bones. This represents a significant limitation, as the optical and biological properties of living tissues can differ considerably from post-mortem tissues. Living biological tissues contain a range of chromophores such as water, oxyhemoglobin (HbO2), deoxyhemoglobin (Hb), myoglobin, melanin, cytochromes, flavins, and water molecules. These chromophores can significantly influence the absorption, scattering, and reflection of light, potentially altering the light penetration results observed in our cadaveric samples.

## Conclusion

Furthermore, different light wavelengths have varying degrees of absorption and scattering in biological media and tissues, suggesting that direct extrapolation of our findings to clinical scenarios may require further evaluation. In particular, the interaction of light with the chemical and mineral composition of living bone, as well as its constant remodeling, may have significant implications for PBM efficacy not fully reflected in our cadaver-based model.

Therefore, while our findings provide valuable insights and suggest the potential for standardizing parameters in PBM based on bone density and thickness, it is crucial to recognize that these results should be interpreted with caution. Future research should focus on studying the interaction of PBM with living bone tissues and consider the influence of chromophores and bone's chemical composition on light penetration.

## Data Availability

The data supporting the findings of this research are available upon request. Access inquiries should be directed to the corresponding author.
